# From Fluid Responsiveness to Prognosis: The Emerging Role of Point-of-Care Echocardiography in Sepsis

**DOI:** 10.3390/diagnostics15202612

**Published:** 2025-10-16

**Authors:** Andrea Piccioni, Gloria Rozzi, Giacomo Spaziani, Michela Novelli, Mariella Fuorlo, Marcello Candelli, Giulia Pignataro, Luca Santarelli, Marcello Covino, Antonio Gasbarrini, Francesco Franceschi

**Affiliations:** 1 Department of Emergency Medicine, Fondazione Policlinico Universitario Agostino Gemelli IRCCS, 00168 Rome, Italymarcello.candelli@policlinicogemelli.it (M.C.); giulia.pignataro@policlinicogemelli.it (G.P.);; 2 Department of Emergency Medicine, Università Cattolica del Sacro Cuore of Rome, 00168 Rome, Italymichelanovelli5@gmail.com (M.N.); 3 Department of Translational Medicine and Surgery, Fondazione Policlinico Universitario Agostino Gemelli IRCCS, Università Cattolica del Sacro Cuore di Roma, 00168 Roma, Italy

**Keywords:** echocardiography and sepsis, sepsis, fluid responsiveness, stroke volume, GLS and sepsis, echocardiography and prognosis in sepsis

## Abstract

Sepsis is a life-threatening condition that requires early recognition and intervention to improve patient outcomes. Optimizing hemodynamic management is crucial, and clinicians must utilize all available tools to guide therapy effectively. Echocardiography is a rapid, non-invasive, and repeatable method that has emerged as a valuable tool in the management of septic patients. Studying its role can provide insights into both therapeutic guidance and prognostic assessment. The primary aim of this review is to highlight the importance of echocardiography in the hemodynamic management of patients with sepsis. The secondary objective is to assess its prognostic value, as echocardiography can inform both the immediate management of critically ill patients and their overall prognosis. A narrative review of the literature published in the last 15 years was conducted using PubMed, and references were managed with Mendeley. Articles focusing on adult and pediatric patients, as well as relevant animal studies, which evaluated echocardiographic assessment of cardiac function, fluid responsiveness, or hemodynamic management were included. Multiple studies demonstrate that echocardiography is a reliable, non-invasive, and easily repeatable tool for assessing fluid responsiveness in septic patients. It allows for dynamic monitoring of stroke volume, VTI, and other hemodynamic parameters, supporting tailored fluid and vasoactive therapy. Additionally, echocardiography provides prognostic insights, with right ventricular dysfunction emerging as a strong predictor of increased mortality. Other parameters, including global longitudinal strain and left ventricular diastolic function, further contribute to risk stratification. Echocardiography is an indispensable tool in the management of sepsis, offering both real-time guidance for hemodynamic optimization and valuable prognostic information. Its routine use can enhance personalized care and improve clinical outcomes in critically ill septic patients.

## 1. Introduction

Sepsis is a life-threatening organ dysfunction resulting from a dysregulated host response to infection [1,2]. Its incidence and severity are higher in the elderly, males, and certain racial and ethnic groups, and are further amplified by socioeconomic disadvantage, geographic factors, and comorbidities [3].

Early recognition remains challenging, making sepsis a leading cause of death in emergency departments [4,5].

Diagnosis is complex. Clinicians rely on scores, such as qSOFA, SIRS, or EWS [6], alongside biomarkers like procalcitonin [7] and presepsin [8,9].

Clinical presentations vary as some patients show classic infection signs, while others—particularly the elderly or immunocompromised—display subtle neurological or systemic symptoms [10]. Septic shock represents the extreme manifestation.

Sepsis triggers a dysregulated inflammatory response, disrupting endothelial, hormonal, metabolic, and immune pathways. These changes drive multi-organ dysfunction, affecting cardiovascular, hematologic, neurological, pulmonary, hepatic, and renal systems [11].

Cardiovascular compromise arises primarily from vasoplegia, relative or absolute hypovolemia, and septic myocardial dysfunction [12].

Endothelial dysfunction mediates inflammation and coagulation via nitric oxide, prostacyclins, and cytokines (e.g., TNF-α, IL-1, IL-6), impairing alpha-adrenergic-receptor sensitivity and vasoconstrictive responses [13].

Vasoplegia promotes relative hypovolemia through peripheral vasodilation, increased capillary permeability, and plasma redistribution, reducing effective intravascular volume and necessitating aggressive fluid therapy [14,15,16].

Myocardial dysfunction, characterized by impaired contractility and often associated with ischemia, contributes significantly to multi-organ failure, driven by cytokine excess, oxidative stress, acidosis, and altered cardiac metabolism [17].

Clinically, it manifests as reduced ejection fraction, elevated ventricular filling pressures, and diminished responsiveness to inotropes.

In this context, point-of-care echocardiography (POC-Echo) has emerged as a rapid bedside tool for real-time assessment of cardiac function. It enables tailored hemodynamic management by guiding fluid resuscitation, vasopressor, and inotropic therapy. Furthermore, echocardiographic parameters provide prognostic insights, helping clinicians identify patients at higher risk of adverse outcomes [18,19].

Given the complexity of sepsis-induced cardiovascular dysfunction and the need for timely intervention, this review explores the role of POC-Echo in guiding treatment decisions and supporting prognostic evaluation in critically ill patients.

## 2. Materials and Methods

This narrative review was conducted by searching PubMed for articles published in the last 15 years using keywords such as “echocardiography and sepsis,” “sepsis and fluid responsiveness,” “stroke volume and sepsis,” “GLS and sepsis,” “echocardiography, prognosis, and sepsis,” and “septic cardiomyopathy.” Studies were initially screened based on titles and abstracts, and full texts were reviewed when relevant. Articles focusing on adult and pediatric patients, as well as animal studies evaluating echocardiographic assessment of cardiac function, fluid responsiveness, or hemodynamic management, were included. Studies published before 2010 were excluded. References were organized using Mendeley, and key data extracted included echocardiographic parameters, fluid management strategies, hemodynamic outcomes, and clinical endpoints such as mortality and vasopressor use.

## 3. Results

### 3.1. Echocardiography-Guided Hemodynamic Management in Sepsis

Echocardiography is a fast, non-invasive, and versatile tool for managing hemodynamics in cases of sepsis and septic shock. It enables continuous monitoring of cardiac function and volume status, helping to identify specific patterns such as sepsis-induced myocardial dysfunction, hypovolemia, or right ventricular alterations—conditions that may not be immediately evident using traditional clinical parameters. The use of echocardiography has been linked to a significant reduction in mortality at 28 days [20], a decrease in the volume of fluids administered, and a quicker discontinuation of vasopressors [21]. Additionally, in patients with sepsis due to trauma, echocardiography-guided monitoring demonstrated a reduction in mortality, with a hazard ratio of approximately 0.83 (95% CI 0.73–0.95) [20].

1. **Preload and fluid responsiveness**
  - **Inferior vena cava (IVC)**: Measurement of diameter and respiratory variations is useful for estimating intravascular volume and the probability of response to fluids.
    Ultrasound-driven fluid resuscitation strategies, particularly those that involve measuring the inferior vena cava (IVC) and conducting passive leg lift tests, have shown effectiveness in reducing the volume of fluids infused, shortening hospital stays, and decreasing the number of days spent in intensive care [22]. However, not all studies indicate a clear benefit concerning mortality rates. For example, a randomized controlled trial (RCT) found that respiratory variation in the diameter of the IVC did not improve survival at 30 days, although it did lead to a decrease in the volume of fluids administered [23,24]. The collapse index of the cava vein is actually a good indicator of the end-diastolic volume index during fluid resuscitation [25]. Nonetheless, ultrasound-guided fluid resuscitation is a useful and practical approach for patients with septic shock during the first seven days after admission. This method is associated with reduced early mortality, lower fluid administration, and a decreased incidence of pulmonary oedema compared to early goal-directed therapy [26].
    In summary, echocardiography is a crucial tool for personalized hemodynamic management, helping refine the administration of fluids and vasoactive support by using IVC parameters.
  - **Stroke volume variation (SVV)**: Change in systolic volume indicates relative hypovolemia and predicts response to fluid therapy. The fluid challenge involves rapidly administering 250–500 mL of fluid in less than 30 min. A 15% increase in stroke volume indicates fluid responsiveness [27].
    During sepsis and septic shock, stroke volume (SV) plays a crucial role in evaluating dynamic hemodynamics and fluid responsiveness, surpassing the limitations of static indices such as central pressure [28].
    SV is a reliable dynamic parameter that allows for the assessment of fluid responsiveness over time. A French observational study revealed that 51.3% of patients who initially respond to fluid therapy maintain this response 30 min after the start of fluid infusion, while only 41.3% exhibit a transient response. This insight is crucial for enabling effective therapy that lasts over time, ultimately enhancing patient outcomes [29].
    A narrative review highlights that, according to the Frank–Starling principle, increasing preload raises SV until optimum volume is reached. Beyond this point, additional fluid infusion may be harmful, leading to fluid overload, tissue oedema, and strain on already impaired cardiac function [30].
    Clinical studies demonstrate that dynamic measures, such as SVV, more accurately predict hemodynamic responses compared to static indices, achieving notable performance in assessing fluid responsiveness, even in ventilated patients. In cases where a septic patient requires mechanical ventilation, these parameters can be utilized to better manage the patient’s hemodynamic and enhance their fluid responsiveness to therapy [31].
    A meta-analysis involving ventilated patients with low tidal volumes (≤8 mL/kg) confirms that SVV has an area under the curve (AUC) of approximately 0.90 for predicting fluid responsiveness. This is further supported by dynamic tests such as the end-expiratory occlusion test and tidal volume challenge, both of which have an AUC of around 0.92 [32].
    An observational study indicated that septic patients who showed an improvement in SV during treatment received, on average, about 350 mL more fluids than those who did not show improvement (1241 mL vs. 893 mL; *p* = 0.018). This suggests that adequate volume expansion may be essential for increasing SV and, consequently, enhancing organ perfusion. Therefore, monitoring SV may serve as an excellent parameter for determining the appropriate volume of fluids to be administered to a patient for achieving genuine improvement [33].
2. **Cardiac output and perfusion**
  **LVOT VTI** (Velocity Time Integral of the left ventricular outflow pathway) is a valuable tool for calculating cardiac output and evaluating tissue perfusion.
  The Velocity Time Integral (VTI), measured at the left ventricular outflow tract (LVOT), is increasingly recognized in the hemodynamic evaluation of patients with sepsis and septic shock due to its ability to noninvasively estimate systolic output and cardiac output. Studies conducted in emergency rooms and intensive care units have demonstrated its feasibility, reproducibility, and clinical usefulness [34].
  VTI has proven to be a reliable predictor of fluid responsiveness [35].
  This is evident through its response to an ultrasound-guided fluid challenge lasting 10 s [36], and by assessing the percentage change in VTI-LVOT after volume loading in patients with septic shock who are on mechanical ventilation [37,38].
  Innovative applications, such as the VTI-VeXUS index, integrate output measurement with the assessment of venous congestion for a more complete hemodynamic evaluation and as a predictor of mortality [39].
  Other studies have illustrated a moderate concordance between VTI-LVOT and alternative measures of cardiac output, such as those obtained from the carotid artery. Nonetheless, VTI is considered superior to these alternative methods, increasing its relevance in emergency contexts [40]. Furthermore, point-of-care ultrasound has shown a significant clinical impact of VTI in assessing volume responsiveness and circulatory function in patients with septic shock, especially when compared to variations in the inferior vena cava diameter [41]. Overall, VTI is emerging as a dynamic and versatile parameter, supported by growing evidence that underscores its importance for personalizing fluid therapy [42], and optimizing tissue perfusion in sepsis, as well as for critically ill patients in general [38,43].
3. **Serial Monitoring**
  Serial monitoring entails performing repeated echocardiographic assessments over time [44]. This approach allows us to track changes in cardiac function, highlighting potential prognostic implications [45], the effectiveness of fluid therapy, and the response to vasoactive drugs. It enables a personalized management strategy for sepsis.
  As demonstrated by the observational analysis conducted by Geri et al. (2019) [46], integrating echocardiographic parameters with clinical data can help identify different cardiovascular phenotypes in septic shock, thereby enhancing the personalization of therapeutic strategies. Specifically, echocardiography can distinguish patients for whom preload is the primary determinant of cardiac output from those in which myocardial dysfunction is dominant. This differentiation aids in deciding between volume expansion and inotropic support [46].
  In this context, Vignon (2020) underscores the importance of serial assessment over simple continuous monitoring of cardiac output, as echocardiography incorporates qualitative elements concerning ventricular function and cardiac filling [47].
  Recent literature indicates that echocardiography provides more comprehensive and contextualized information compared to cardiac flow monitors. This added detail is not only beneficial for predicting the response to fluids but also for guiding the timing and indications for utilizing inotropes [48].
  Overall, echocardiography stands out as a central and multimodal tool, capable of addressing the limitations of standard monitoring and supporting a tailored strategy in managing septic shock (Table 1).

### 3.2. Pathophysiology of Septic Cardiomyopathy

Sepsis-induced cardiomyopathy (SIC) is a transient but clinically significant syndrome characterized by impaired left ventricular (LV) systolic and diastolic function, right ventricular (RV) dysfunction, and reduced myocardial strain. The underlying pathophysiology is multifactorial. Inflammatory mediators, including TNF-α, IL-1β, and IL-6, play a central role, leading to nitric oxide overproduction, mitochondrial dysfunction, impaired calcium handling, and autonomic dysregulation, all of which contribute to reversible myocardial depression [49,50,51].

Microvascular dysfunction and altered ventricular loading conditions further impair cardiac output and oxygen delivery, amplifying tissue hypoperfusion [52]. Experimental studies in animal models have confirmed these mechanisms and have demonstrated echocardiographic correlates that mimic human disease, providing a translational basis for clinical observations [53,54,55].

The clinical presentation of SIC is highly heterogeneous. Patients with multiple comorbidities, particularly elderly individuals or those with cardiovascular risk factors, are more likely to develop diastolic dysfunction of the left ventricle and typically have elevated troponin levels. In contrast, patients experiencing right ventricular dysfunction tend to show higher values of the SOFA score and NT-proBNP [56,57]. LV ejection fraction (LVEF) may remain preserved despite significant myocardial impairment, particularly in patients with concurrent RV dysfunction or diastolic abnormalities [58]. Furthermore, pre-existing cardiac dysfunction has been shown to predispose patients to worse outcomes during sepsis, highlighting the importance of baseline cardiac assessment [59].

### 3.3. Prognostic Role of Echocardiography

Echocardiography is a cornerstone in the prognostic evaluation of septic patients. Early identification of myocardial dysfunction allows risk stratification and guides hemodynamic management [52,60,61]. The global longitudinal strain (GLS), measured using speckle-tracking echocardiography, is a sensitive indicator of left ventricular systolic function. In cases of sepsis, GLS has been found to be both earlier and more accurate than ejection fraction (EF) in detecting subclinical myocardial dysfunction. Reduced GLS values are associated with greater clinical severity, a higher need for vasopressor support, and increased mortality rates [62,63,64,65]. Therefore, GLS is an effective tool for prognostic assessment and risk stratification in septic patients [66].

Although LV systolic dysfunction has shown variable associations with mortality, LV diastolic dysfunction consistently predicts adverse outcomes, including prolonged ICU stay and increased mortality [67,68].

RV dysfunction has emerged as one of the strongest echocardiographic predictors of short- and long-term mortality, independent of LV function [56,57,69,70,71,72,73,74]. It is associated with 30 days mortality even in mechanically ventilated patients [75]. In a meta-analysis including 1373 patients, RV dysfunction was present in about one-third of cases and was associated with increased need for renal replacement therapy and with both higher short- and long-term mortality [76].

Key RV parameters, including tricuspid annular plane systolic excursion (TAPSE) and RV strain, provide robust prognostic information and have been validated across multiple cohorts and systematic reviews [77,78,79].

In a cohort of 104 patients, right ventricular dysfunction—defined as TAPSE <16 mm and RV FAC < 35%—was found to be a predictor of 28-day in-hospital mortality, and was further associated with elevated lactate levels and prolonged ICU length of stay [70].

Numerous studies indicate that echocardiograms are more reliable predictors of mortality in sepsis patients compared to troponin levels alone. While troponin primarily indicates acute myocardial damage and can be elevated due to non-ischemic factors, echocardiography directly assesses ventricular function and the hemodynamic changes caused by the systemic inflammatory response [80,81,82]. This makes echocardiograms more closely associated with prognosis and risk stratification in septic patients.

Serial echocardiography is recommended over a single assessment, as SIC is dynamic and reversible. Repeated measurements allow monitoring of cardiac recovery and response to therapeutic interventions and it can identify patients with persistent RV or biventricular dysfunction, who are at particularly high risk for mortality [83,84,85,86]. Integrating echocardiography into routine management of sepsis therefore offers both mechanistic insights and prognostic guidance (Table 2), reinforcing its role as an essential tool in critical care [86,87,88].

### 3.4. Comparison Between POC-Echo and Invasive Hemodynamic Monitoring in Sepsis

Recent evidence also highlights the potential role of invasive hemodynamic monitoring in selected septic patients. In particular, the early use of pulmonary artery catheterization in cases complicated by sepsis-associated cardiogenic shock has been associated with lower in-hospital mortality and increased utilization of advanced circulatory support strategies [89]. In this context, echocardiography can be used as a screening tool to identify septic patients with a potential cardiogenic component. In such cases, a pulmonary artery catheter (PAC) may be useful for continuous invasive monitoring [90]. However, invasive hemodynamic monitoring in septic patients requires an intensive care environment, is a delicate procedure, and is impractical to perform in the emergency department or in the emergency room setting. Moreover, it demands highly advanced technical skills, which not all clinicians possess.

Echocardiography remains an extremely valuable and effective tool in the emergency management of sepsis, particularly for the rapid assessment of hemodynamic status. However, in selected clinical scenarios, invasive monitoring using a PAC may also be warranted. Importantly, these two approaches are not mutually exclusive; one does not replace the other. Rather, they can be complementary, with echocardiography providing an initial, noninvasive assessment of the patient’s hemodynamic condition, while invasive monitoring may be reserved for more severe or complex cases where detailed, continuous data are clinically necessary. Although current evidence remains largely observational, these findings suggest that combining noninvasive and invasive modalities can enhance clinical decision-making and potentially improve patient outcomes.

### 3.5. Clinical Relevance of Echocardiography in Early Sepsis Management vs. Accuracy of Cardiac Magnetic Resonance (CMR)

Cardiac Magnetic Resonance Imaging (MRI) has emerged as a crucial tool in the investigation of sepsis and septic shock with associated cardiomyopathy, given its ability to provide detailed structural and functional information. In large animal models, septic animals compared to controls developed significant impairment of left ventricular function, including reduced ejection fraction, strain, and ventricular–arterial coupling, changes that were not explained by differences in preload, afterload, or heart rate. CMR has also documented myocardial alterations that closely resemble those seen in humans, showing that ventricular dysfunction may occur independently of microvascular ischemia or catecholamine surges [91].

In both human and animal septic shock, reductions in ejection fraction and progressive increases in end-diastolic volumes have been observed, with normalization within 10 days in survivors. Cardiac dysfunction appears to be primarily linked to edema, wall thinning, and loss of ventricular mass, rather than hemodynamic loading conditions. In non-survivors, by contrast, the absence of an increase in end-diastolic volume and the presence of more severe early diastolic dysfunction correlate with poor outcome [92].

In clinical studies of critically ill patients, CMR has revealed a heterogeneous septic cardiomyopathy phenotype, with reduced systolic function, abnormal strain, and ventricular remodeling, offering superior characterization compared with echocardiography [93]

Collectively, these findings support the role of CMR as a key non-invasive modality to elucidate the pathophysiology of septic cardiomyopathy, validate experimental models, and inform prognosis and therapeutic strategies. However, echocardiography remains the superior tool in the early management of sepsis, given its widespread availability, lower cost, and immediate applicability at the bedside. CMR, in contrast, is limited by restricted access in emergency settings, high cost, the need for specialized expertise, long waiting lists, and the additional delays caused by infection-control protocols in potentially contagious patients. Thus, while CMR provides unparalleled insights into cardiac structure and function, echocardiography continues to be the most useful non-invasive modality for guiding hemodynamic assessment and therapeutic management in septic patients. Future studies should aim to clarify the true clinical contribution of CMR in sepsis.

## 4. Discussion

This review highlights the pivotal role of POC-Echo in both hemodynamic management and prognostic evaluation of septic patients. Dynamic echocardiographic parameters, including SV, SVV, and LVOT VTI, reliably predict fluid responsiveness, enabling tailored fluid resuscitation and vasoactive therapy [33,94].

Our findings align with prior observational studies and meta-analyses demonstrating that echocardiographic evaluation of RV function is a powerful prognostic tool in sepsis. RV dysfunction has consistently been associated with both short- and long-term mortality, independent of left ventricular performance [71,73,80]. Unlike static measures such as central venous pressure (CVP), which provide only a snapshot of intravascular volume and have shown limited predictive accuracy, echocardiography allows dynamic, real-time assessment of ventricular function and hemodynamic responsiveness. Similarly, conventional biomarkers like troponin primarily reflect myocardial injury but do not capture functional impairment or the complex hemodynamic derangements characteristic of sepsis [80,82].

By directly visualizing RV performance—including parameters such as TAPSE, RV FAC, and RV strain—echocardiography provides a more sensitive and specific evaluation of cardiac compromise [77,78]. This dynamic insight not only enables earlier identification of high-risk patients but also guides clinical decision-making regarding fluid resuscitation and vasoactive therapy. For example, detecting RV dysfunction can inform individualized fluid management strategies, preventing both under- and over-resuscitation, and help tailor vasopressor support to optimize cardiac output and tissue perfusion. In this way, echocardiography bridges prognostic assessment with therapeutic guidance, reinforcing its superior value over static measures or biomarker-based evaluations and highlighting its role as a key tool in improving patient-centered outcomes in sepsis.

The strength of this review lies in the wide range of clinical studies considered, including adult, paediatric, mechanically ventilated patients, those with comorbidities such as diabetes, and even animal models, providing robust scientific support for the findings. Furthermore, a substantial proportion of the evidence derives from high-impact meta-analyses and systematic reviews, most of which have been published within the last five years, underscoring both the robustness and contemporary relevance of the available data.

Importantly, the clinical utility of echocardiography is underscored: an experienced clinician can integrate echocardiographic assessment with laboratory and instrumental tests during bedside evaluation, enabling the development of standardized protocols in which POC-Echo plays a central role in sepsis therapy.

Given the life-threatening nature of sepsis, every available tool that aids in management and improves outcomes is invaluable.

However, several limitations must be acknowledged. Echocardiography remains operator-dependent, introducing potential biases. This emphasizes the need for emergency physicians—the first responders in sepsis—to acquire comprehensive skills in echocardiography, ensuring accurate interpretation and optimal patient care.

Moreover, not all hospitals have the resources to equip their emergency departments with echocardiography devices, which restricts access to this valuable diagnostic tool. In addition, structured training programs and certification courses can be expensive, creating further barriers for institutions and practitioners. Even when training is available, achieving proficiency requires years of consistent practice, making the learning curve particularly steep. Finally, echocardiography is inherently subject to personal interpretation; as a result, evaluations may vary between operators and occasional errors in clinical judgment remain inevitable. These constraints highlight the importance of both systematic training and cautious integration of echocardiography into routine sepsis management.

Anyway, the clinical implications are significant. Integration of POC-Echo into sepsis management protocols facilitates individualized hemodynamic optimization, reduces the risk of fluid overload, and enables earlier recognition of myocardial dysfunction. By providing real-time, bedside assessment, echocardiography can improve decision-making regarding fluid therapy, inotropic support, and vasopressor titration, ultimately contributing to improved patient outcomes and resource utilization.

Future research should focus on standardizing POC-Echo protocols and conducting multicentre trials to evaluate its impact on patient-centered outcomes, including mortality, organ failure, and ICU length of stay. Such studies will help define optimal strategies for echocardiography-guided management and strengthen its role in early sepsis care.

In conclusion, POC-Echo represents an indispensable tool in the management of septic patients. By guiding fluid and vasoactive therapy, detecting cardiac dysfunction, and providing prognostic information—particularly through RV assessment and GLS measurement—it enhances individualized care and supports timely, evidence-based clinical decisions.

## 5. Conclusions

Echocardiography is a powerful, non-invasive tool that should become a standard component of sepsis management. By guiding fluid therapy, detecting cardiac dysfunction, and providing prognostic insights—especially through right ventricular assessment—it enables more precise and timely interventions. Integrating echocardiography into emergency and critical care protocols has the potential to improve outcomes and save lives, making it an indispensable tool in modern sepsis care. Future studies should assess standardized point-of-care echocardiography protocols for managing sepsis.

## Figures and Tables

**Table 1:** Comparative Table about the role of echocardiography in fluid management in sepsis. *Symbol legend:* ↓ denotes a decrease or reduction (for example, in mortality, incidence, or other measured outcomes).

Reference	Year	Study Type	Population/Model	Echocardiographic Focus	Prognostic Implications
**Chen Z et al. [22]**	2024	Systematic review & meta-analysis	Patients with septic shock	Ultrasound-guided fluid resuscitation (IVC, PLR)	↓ mortality, ↓ infused fluids, ↓ hospital/ICU stay
**Musikatavorn K et al. [23]**	2021	Randomized Controlled Trial	202 patients, sepsis/septic shock	Point-of-care ultrasound vs. clinical management	Reduced infused fluids, no clear survival benefit
**Yan Z et al. [24]**	2020	Clinical study	40 patients with septic shock	IVC diameter + lung ultrasound B-lines	Better assessment of fluid responsiveness, ↓ risk of fluid overload
**Zhao J & Wang G [25]**	2016	Observational	42 septic shock patients	IVC collapsibility index vs. GEDVI, CI, CVP	IVCCI correlated with GEDVI; useful non-invasive index of intravascular volume
**Yuan J et al. [26]**	2020	Systematic review	Septic shock patients	Ultrasound vs. Early Goal-Directed Therapy (EGDT)	↓ early mortality (7 d), ↓ fluids, ↓ pulmonary edema; no benefit at 28 days mortality
**Martin GS et al. [27]**	2020	Consensus statement (POQI)	Perioperative/critically ill patients	Fluid responsiveness & venous capacitance	Defines concepts and recommends dynamic ultrasound assessment
**Kenny JÉS et al. [28]**	2024	Prospective observational pilot	Patients in early resuscitation	Simultaneous venous-arterial Doppler (Starling curve)	Feasibility study; proposes a novel hemodynamic index
**Roger C et al. [29]**	2019	Observational (FCREV study)	143 Septic patients	Fluid challenge + echocardiography	Fluid responsiveness is dynamic and changes over time
**Latham H et al. [33]**	2023	Observational (abstract, CCM)	Sepsis/shock patients	Infused fluid volume vs. stroke volume change	Early fluid volume may predict hemodynamic response
**Parker CW et al. [35]**	2022	Review/Educational	Emergency Department	VTI (Velocity-Time Integral)	VTI useful for bedside stroke volume and CO assessment
**Wu Y et al. [36]**	2014	Clinical study	Septic patients	10-second fluid challenge + TTE (VTI-LVOT)	Change in VTI predicts fluid responsiveness
**Wang J et al. [37]**	2020	Clinical study	Septic shock patients	VTILVOT variation rate	Useful for assessing fluid responsiveness
**Sasidharan P et al. [38]**	2025	Clinical study	Sepsis-related acute circulatory failure	LVOT VTI	LVOT VTI predictive of fluid responsiveness
**Prager R et al. [39]**	2025	Exploratory observational	Septic shock patients	VTI-VeXUS index (novel hemodynamic marker)	Promising tool to stratify congestion and response
**Chanthawatthanarak S et al. [40]**	2025	Prospective study	ED septic shock patients	Carotid vs. LVOT cardiac output (non-invasive)	Good correlation; feasible alternative method, VTI better performance
**Saji SZ et al. [42]**	2025	Systematic review	Adults with sepsis/septic shock	LVOT VTI	LVOT VTI is accurate for fluid management
**Blanco P [43]**	2020	Review	Critically ill patients	VTI & stroke volume	Provides rationale for using VTI as reliable bedside measure
**Soliman-Aboumarie H et al. [44]**	2021	Review	ICU patients	Multiparametric echocardiography	Essential ICU tool for diagnosis and decision-making
**de Braga Lima Carvalho Canesso M et al. [45]**	2019	Clinical study	50 septic patients	Speckle-tracking echocardiography (GLS)	GLS changes correlated with dysfunction and prognosis
**Suh GJ et al. [48]**	2023	Narrative review	Septic shock	Hemodynamic management beyond SSC guidelines	Echo central to advanced hemodynamic strategies

**Table 2:** Comparative Table of Clinical Studies about prognostic role of echocardiography. *Symbol legend:* ↑ denotes an increase (for example, in mortality, incidence, or other measured outcomes).

Reference	Year	Study Type	Population/Model	Echocardiographic Focus	Prognostic Implications
**Carbone F, Liberale L, et al. [49]**	2022	Narrative Review	Human/experimental	General echo in SCM	Describes diagnostic & prognostic potential
**Hasegawa D, Ishisaka Y, et al. [52]**	2023	Systematic review/meta-analysis	23 studies, >2000 patients	LV/RV dysfunction	SCM associated with ↑ mortality
**Ince ME, Turgut K, et al. [53]**	2019	Observational	Dogs	Tissue Doppler (systolic/diastolic)	Abnormal indices predicted worse prognosis
**Lu N-F, Niu H-X, et al. [56]**	2024	Clinical study	Septic patients (~184)	LV vs. RV vs. biventricular	Phenotypes linked to different prognoses-RV dysfunction ↑ mortality
**Ravikumar N, Sayed MA, et al. [58]**	2021	Review	Human	LV/RV dysfunction	Presence of SIC ↑ mortality
**El Mokadem M, El Maraghi S, et al. [60]**	2024	Clinical observational	50 septic patients	LV strain, biomarkers	GLS predictor of in-hospital mortality
**Tucker RV, Williams K, et al. [61]**	2022	Observational	110 ED septic patients	Focused cardiac US	SIC is associated with increased 90-day mortality.
**Yu J, Zheng R, et al. [63]**	2022	Cohort study	124 Septic patients	LV-GLS	LV-GLS predicted LV systolic dysfunction & death
**Innocenti F, Palmieri V, et al. [64]**	2018	Observational	Septic patients (~200)	LV systolic-GLS	GLS predicts short-term outcomes, independent of SOFA
**Vallabhajosyula S, Rayes HA, et al. [65]**	2019	Systematic review	Septic patients (120 studies)	Speckle-tracking echocardiography (STE)	STE strong predictor of mortality
**Zhao JL, Wang R, et al. [66]**	2023	Observational	58 Septic patients	RV echo + GLS + biomarkers	GLS + biomarkers predicted mortality
**Thockchom N, Bairwa M, et al. [68]**	2023	Longitudinal study	132 patients Sepsis + diabetes	LV diastolic	Diastolic dysfunction predicted worse outcome
**Lin YM, Lee MC, et al. [69]**	2022	Meta-analysis	>20 studies	LV/RV	SICM linked with increased mortality
**Koowattanatianchai S, Kochaiyapatana P, et al. [70]**	2025	Prognostic analysis	104 Septic patients	RV systolic	RV dysfunction predicted short-term survival
**Lanspa MJ, Cirulis MM, et al. [71]**	2021	Cohort study	393 Septic shock patients	RV parameters	RV dysfunction associated with worse outcomes
**Innocenti F, Palmieri V, et al. [72]**	2020	Observational	252 ED septic patients	RV systolic	RV dysfunction frequent, prognostic role
**Kim JS, Kim YJ, et al. [73]**	2020	Observational	778 Septic shock patients	RV function	RV dysfunction linked with mortality
**Vallabhajosyula S, Kumar M, et al. [74]**	2017	Historical cohort (8 years)	388 ICU septic patients	RV dysfunction	RV dysfunction independently predicted mortality
**Zhang H, Huang W, et al. [75]**	2021	Observational	215 Ventilated septic pts	Various RV dysfunction types	RV failure and RV dysfunction are associated with 30 days mortality
**Vallabhajosyula S, Shankar A, et al. [76]**	2021	Meta-analysis	1373 patients	RV dysfunction	RV dysfunction ↑ short- & long-term mortality
**Perencin A, Curreri C, et al. [77]**	2025	Systematic review/meta-analysis	1812 Septic/septic shock patients	TAPSE	TAPSE predictive of mortality
**Liu H, He H, et al. [78]**	2025	Retrospective cohort	93 SCM patients	TAPSE	Low TAPSE predicted poor outcome
**Sanderson T, Samuels T. et al. [79]**	2025	Cohort study	ICU septic patients	RV strain, myocardial work	Both associated with mortality
**Sukrisd Koowattanatianchai et al. [70]**	2025	prospective cohort study	104 adult septic patients	RV dysfunction	RV dysfunction ↑ mortality
**Innocenti F, Palmieri V, et al. [80]**	2022	Observational	Septic patients	Echo vs. troponin	Echo predicts the short- and medium-term mortality rate
**Gajardo AIJ, Ferrière-Steinert S, et al. [81]**	2025	Systematic review/meta-analysis	6242 patients from 17 studies	Biomarkers (not echo)	Elevated troponin not predicted higher mortality in sepsis
**Wang TT, Jiang L. [82]**	2017	Observational	125 Critically ill septic patients	Biomarker study	hs-cTnT not associated with severity of sepsis
**Innocenti F, Palmieri V, et al. [85]**	2021	Observational	Septic shock pts	Speckle tracking	LV and RV dysfunction

## Data Availability

The original contributions presented in this study are included in the article. Further inquiries can be directed to the corresponding authors.

## Abbreviations

The following abbreviations are used in this manuscript:

POC-Echo	Point-of-Care Echocardiography
qSOFA	quick Sequential Organ Failure Assessment
SIRS	Systemic Inflammatory Response Syndrome
EWS	Early Warning Score
SV	Stroke Volume
SVV	Stroke Volume Variation
LVOT	Left Ventricular Outflow Tract
VTI	Velocity Time Integral
VTI-LVOT	Velocity Time Integral measured at the Left Ventricular Outflow Tract
VTI-VeXUS	VTI-Venous Excess Ultrasound Score
IVC	Inferior Vena Cava
RCT	Randomized Controlled Trial
LV	Left Ventricle
RV	Right Ventricle
LVEF	Left Ventricular Ejection Fraction
TAPSE	Tricuspid Annular plane systolic excursion
MRI	Magnetic Resonance Imaging
CMR	Cardiac Magnetic Resonance
